# A systematic SNP selection approach to identify mechanisms underlying disease aetiology: linking height to post-menopausal breast and colorectal cancer risk

**DOI:** 10.1038/srep41034

**Published:** 2017-01-24

**Authors:** Rachel J. J. Elands, Colinda C. J. M. Simons, Mona Riemenschneider, Aaron Isaacs, Leo J. Schouten, Bas A. Verhage, Kristel Van Steen, Roger W. L. Godschalk, Piet A. van den Brandt, Monika Stoll, Matty P. Weijenberg

**Affiliations:** 1 Department of Epidemiology, GROW–School for Oncology and Developmental Biology, Maastricht University, Maastricht, the Netherlands; 2 Institute of Human Genetics, Genetic Epidemiology, University of Münster, Münster, Germany; 3 Department of Bioinformatics, Straubing Center of Science, Straubing, Germany; 4 Department of Epidemiology, Genetic Epidemiology Unit, Erasmus Medical Center, Rotterdam, the Netherlands; 5 Department of Biochemistry, Maastricht Centre for Systems Biology (MaCSBio), CARIM–School for Cardiovascular Diseases, Maastricht University, Maastricht, the Netherlands; 6 Department of Electrical Engineering and Computer Science, Montefiore Institute, University of Liège, Liège, Belgium; 7 Department of Pharmacology and Toxicology, NUTRIM–School of Nutrition and Translational Research in Metabolism, Maastricht University, Maastricht, the Netherlands

## Abstract

Data from GWAS suggest that SNPs associated with complex diseases or traits tend to co-segregate in regions of low recombination, harbouring functionally linked gene clusters. This phenomenon allows for selecting a limited number of SNPs from GWAS repositories for large-scale studies investigating shared mechanisms between diseases. For example, we were interested in shared mechanisms between adult-attained height and post-menopausal breast cancer (BC) and colorectal cancer (CRC) risk, because height is a risk factor for these cancers, though likely not a causal factor. Using SNPs from public GWAS repositories at *p*-values < 1 × 10^−5^ and a genomic sliding window of 1 mega base pair, we identified SNP clusters including at least one SNP associated with height and one SNP associated with either post-menopausal BC or CRC risk (or both). SNPs were annotated to genes using HapMap and GRAIL and analysed for significantly overrepresented pathways using ConsensuspathDB. Twelve clusters including 56 SNPs annotated to 26 genes were prioritised because these included at least one height- and one BC risk- or CRC risk-associated SNP annotated to the same gene. Annotated genes were involved in Indian hedgehog signalling (*p*-value = 7.78 × 10^−7^) and several cancer site-specific pathways. This systematic approach identified a limited number of clustered SNPs, which pinpoint potential shared mechanisms linking together the complex phenotypes height, post-menopausal BC and CRC.

Knowledge on single nucleotide polymorphisms (SNPs) and gene-environment interactions associated with complex diseases provides insights into underlying etiologic mechanisms^1^,^2^. Genome-wide gene-environment interaction studies have typically been applying two-step approaches that are aimed at increasing power. Two-step genome-wide gene-environment interaction studies often utilise a SNP reduction step, in which the number of SNPs to include in the analysis is reduced^3^. The SNPs are subsequently tested for interaction, limiting multiple testing. However, for large-scale epidemiological studies with exhaustive bio-samples from which DNA is not immediately suitable for genome-wide platforms, *e*.*g*. DNA from nails, the only option is platforms allowing genotyping of a limited number of SNPs. For example, we have previously genotyped toenail DNA using the Agena Bioscience^TM^ MassARRAY^®^ platform, which allows genotyping of a maximum of 40 SNPs at once in large-scale epidemiologic studies^4^. Therefore, an alternative systematic strategy is needed to reduce the number of relevant SNPs for studying disease aetiology through, for example, gene-environment interactions. Data from genome-wide association studies (GWAS) suggest that SNPs associated with complex diseases or traits are not randomly distributed across the genome but tend to co-segregate in regions of low recombination, harbouring functionally linked gene clusters^5^. Such an enrichment of loci associated with complex traits or diseases has been observed throughout the human genome^5^ and offers an opportunity to SNP reduction.

Approaches for gene-environment interaction studies differ according to study objective. Searching for genetic causes of disease is nowadays generally an agnostic approach. In gene-environment-wide interaction studies, the starting point is also typically the genetic variation and how its interaction with the environment can contribute to the missing heritability^6^. Alternatively, studies aimed at understanding how the environment is associated with cancer risk are generally performed via a hypothesis-driven approach where the starting point is the environmental factor and the genetic variation is a time-independent biomarker of pathway involvement^2^. We were interested in the association between adult-attained height and cancer risk. Adult-attained height is an established risk factor for cancer risk at several sites; the most convincing evidence has been reported for post-menopausal breast cancer and colorectal cancer risk^7^,^8^. For every 5 cm increase in height, post-menopausal breast cancer risk is reported to be increased by 7 to 11%^7^,^9^,^10^ and colorectal cancer risk is increased by 6 to 11% in women and 4 to 9% in men^8^,^10^,^11^. Adult-attained height in itself is probably not causally related to cancer, but rather a consistent marker for shared mechanisms determining both height and cancer risk, *e*.*g*. growth processes, which are influenced by factors such as growth promoting hormones and energy balance in early life^12^. Height is determined in the first 20 years of life by aggregated genetic^13^ and environmental components^12^, which determine linear growth but may also spur neoplastic growth later in life. Although adult-attained height may not be a target for intervention to reduce cancer risk, understanding how height is associated with cancer risk is essential to expand our knowledge concerning the pathways that lead to cancer development later in life. To study shared mechanisms between height and post-menopausal breast and colorectal cancer risk, we have applied a systematic SNP reduction strategy based on existing GWAS repositories and based on the fact that SNPs associated with complex diseases or traits tend to co-segregate in regions of low recombination. This knowledge was taken forward and we sought for clusters that included both height- and either postmenopausal breast cancer- or colorectal cancer-associated SNPs (or both) by comprehensively overlaying GWAS for these endpoints.

## Methods

### Search strategy

SNPs from the publically available manually curated National Human Genome Research Institute (NHGRI) Catalog of published GWAS^14^ and the Johnson and O’Donnell database^15^ associated with either height, post-menopausal breast or colorectal cancer risk were selected if these had a *p*-value < 1 × 10^−5^, a minor allele frequency (MAF) ≥1% in Caucasians, and were added to the catalogues up to June, 2014. Selected SNPs also included SNPs from meta-analyses on GWAS, which may have included SNPs with a *p*-value < 1 × 10^−5^ that did not reach this threshold in individual GWAS. The *p*-value cut-off for the selection of SNPs is a rather liberal value given the focus on genetic variation that tags mechanisms important for the multiple phenotypes of interest, in this case, height, post-menopausal breast cancer and colorectal cancer. Therefore, allowing a liberal *p*-value threshold permits one to identify clustered GWAS SNPs for a combination of different traits or diseases rather than clustered GWAS SNPs for a single phenotype. Genome-wide significant common variants (*p*-value < 5 × 10^−8^) and common variants that do not reach this criterion explain substantially large amounts of the heritability of complex traits and complex diseases; because SNPs below genome-wide significance (*p*-value > 5 × 10^−8^) with marginal individual effect sizes may likely interact with other common SNPs and environmental components^16^,^17^. SNPs identified in non-Caucasian populations were included if the corresponding MAF was ≥1% in Caucasians, for the reason that SNP-phenotype associations from different ancestries in independent GWAS might be informative to single out regions that link height to cancer risk. Including these SNPs from GWAS with other ancestries will also make our selection more comprehensive given that a number of SNPs may not yet have been explored in populations from Caucasian ancestry as a consequence of low signal resolution in older GWAS or because of differences in SNP coverage across genotyping platforms.

### Clustering methodology

**Figure 1:**
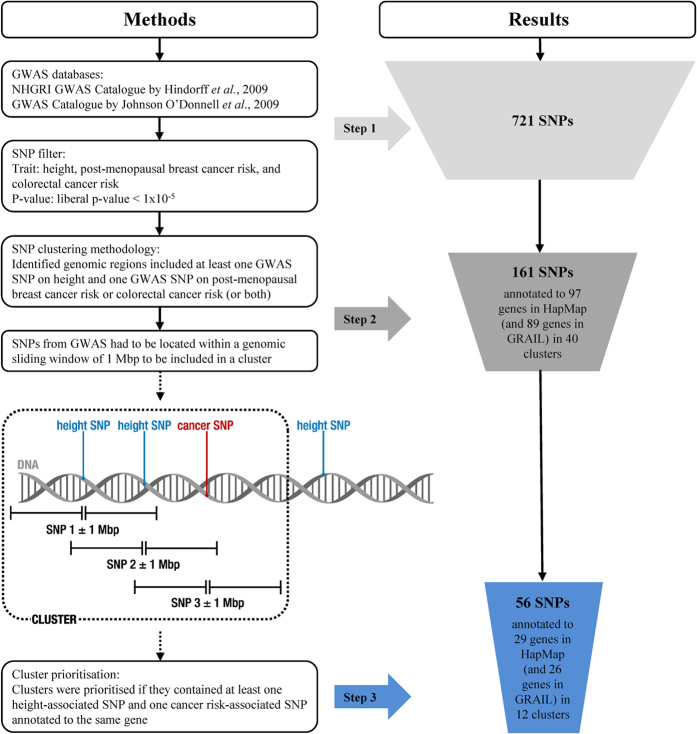
Flow diagram with overview of SNP selection methodology and the corresponding results.

Our clustering methodology was based on the assumption that GWAS SNPs associated with complex diseases or traits are not randomly distributed across the genome but tend to cluster in regions of low recombination^5^. Using a sliding window of 1 megabase pair (Mbp), genomic regions including at least one SNP from GWAS associated with height and one SNP from GWAS associated with either post-menopausal breast or colorectal cancer risk (or both) located within were designated as a SNP cluster. SNPs were clustered from the first height- or cancer risk-associated SNP that was identified from GWAS until no additional SNPs within the genomic sliding window of 1 Mbp could be found (Fig. 1). Each cluster was assigned a unique cluster ID. The reason for implementing a relatively wide-ranging genomic sliding window (1 Mbp) was to allow for a sufficient number of SNPs, associated with multiple phenotypes, to cluster in regions of low recombination. We experimentally tested more conservative genomic sliding windows (0.1, 0.2, 0.3, and 0.5 Mbp), which resulted in identifying clusters with height- and breast cancer risk- or colorectal cancer risk-associated SNPs, but SNPs annotated to the same gene were not always in the same cluster anymore (which particularly affected large clusters with multiple SNPs annotated to the same gene). Furthermore, a few clusters were no longer identified. A wide-ranging genomic sliding window is preferable because the majority of GWAS SNPs reside in non-coding regions, potentially marking long-ranging disease-associated areas rather than pointing to individual genes. For example, 40.8% of SNPs from GWAS in DNAse I hypersensitive sites can be linked to target promotors over distances longer than 250 Kbp^18^.

SNPs from the clusters were geographically mapped to a gene according to HapMap release 37 and annotated to a gene according to “Gene Relationships Among Implicated Loci” (GRAIL) (https://www.broadinstitute.org/mpg/grail/). GRAIL accounts for the three-dimensional structure of the DNA, resulting in functional annotations. SNP clusters were prioritised when these contained at least one height-associated SNP and one cancer risk-associated SNP that were mapped to the same gene according to the HapMap or GRAIL annotation (or both, allowing that HapMap and GRAIL may yield different annotations) or a combination of HapMap and GRAIL annotations. For each SNP in the prioritised set of clusters, the rs-number, mapped gene, publication information, SNP-phenotype information, the significance of the association, the effect size or beta-coefficient, confidence interval, ancestry and the risk allele (reported in the catalogues and from Ensembl (http://www.ensembl.org)) were collected. Within a cluster, pair-wise linkage disequilibrium (LD) was examined using SNAP version 2.2, (https://www.broadinstitute.org/mpg/snap/). Two or more SNPs in high pair-wise LD, *i*.*e*. r^2^ > 0.7, marked redundant information within the cluster. Within LD pairs, SNPs with the lowest evidence for regulatory function annotation were excluded, but only if the cluster criteria were not violated. Ensembl genome browser was used to determine the genomic region of the SNPs and to identify whether these were localised in a regulatory region^19^. Regulatory functional annotation of SNPs was evaluated using a ranking ranging from 1–6 provided by RegulomeDB (http://www.regulomedb.org/)^20^. The ranking is based on the overlap of existing functional data including annotation to cis-expression quantitative trait loci (cis-eQTLs) and evidence for protein/transcription factor binding. SNPs that were likely linked to the expression of a gene target (cis-eQTLs) were assigned the highest possible ranking, i.e. scores 1a-1f, in RegulomeDB. SNPs that likely only affected protein binding were ranked lower (scores 2–3) and SNPs, for which there was minimal binding evidence (rank 4–6) or for which no evidence was available (score 0) were assigned the lowest evidence for regulatory function in RegulomeDB. The rationale to prioritise SNPs on the basis of regulatory information was derived from the knowledge that a significant number of SNPs associated with quantitative traits and common diseases in GWAS are concentrated in non-coding regulatory DNA sequences, therefore it is likely that regulatory processes underlie the relation between a SNP from GWAS and a phenotype^18^,^21^.

### Biological interpretation: gene set over-representation analyses

The gene annotations for the different SNPs in the resulting prioritised set of clusters, were imported to ConsensusPathDB (http://consensuspathdb.org/)^22^ to conduct gene set over-representation analyses. In these analyses, pathways and gene ontology (GO) categories were tested for over-representation in the uploaded gene set. We primarily based these analyses on functional annotations from GRAIL. Tests were based on the hypergeometric test with a *p*-value cutoff set to 0.01. Multiple testing was accounted for and the *q*-value threshold was set at 0.05. Pathway over-representation analyses and GO-over-representation analyses were performed for all clusters combined as well as separately for clusters including height- and post-menopausal breast cancer risk-associated SNPs and clusters including height- and colorectal cancer risk-associated SNPs.

## Results

An overview of the selection steps and the corresponding output is shown in Fig. 1. The NHGRI Catalog included 1751 curated publications with 11,912 SNPs and the Johnson and O’ Donnell database contained 56,411 SNPs from 118 articles. After selecting SNPs on the basis of the *p*-value (*p* < 1 × 10^−5^) and MAF (≥1% in Caucasians) and filtering out duplicates, due to multiple associations in GWAS, we started clustering with 721 SNPs from both GWAS repositories. 514 SNPs were associated with height, 157 SNPs were associated with post-menopausal breast cancer risk and 50 SNPs were associated with colorectal cancer risk. None of the individual SNPs were associated with multiple phenotypes, *i*.*e*. height, post-menopausal breast cancer risk and/or colorectal cancer risk. Using the clustering method with a genomic sliding window of 1 Mbp, 40 clusters containing altogether 161 SNPs annotated to 97 genes on the basis of HapMap and 89 genes on the basis of GRAIL (9 SNPs could not be annotated) were formed, each including at least one SNP associated with height and one SNP associated either with post-menopausal breast or colorectal cancer risk (see Table S1). No SNP clusters were identified with combinations of SNPs that were associated with height, and both post-menopausal breast and colorectal cancer risk.

Twelve clusters containing altogether 56 SNPs, annotated to a total of 29 genes in HapMap and 26 genes in GRAIL (five SNPs could not be annotated), were prioritised as these clusters contained at least one height-associated SNP and one cancer risk-associated SNP that were annotated to the same gene. HapMap and GRAIL SNP-gene annotations were the same for 64.7% of the cases where both annotations were available (n = 51). Characteristics of the SNPs in the 12 prioritised SNP clusters are shown in Tables 1 and S1. Eight SNPs in five of the prioritised clusters were eliminated from the total of 56 SNPs, leading to 48 SNPs in the prioritised clusters, due to the fact that these SNPs were in high LD (r^2^ > 0.7) with another SNP in the same cluster, therefore these SNPs were likely to tag redundant information. Of the 12 prioritised clusters, 8 clusters included 19 height- and 14 post-menopausal breast cancer risk-associated SNPs and four clusters included 10 height- and five colorectal cancer-risk associated SNPs. Of the 33 SNPs in height-breast cancer clusters, 26 SNPs were annotated to the same gene in sets of two or more height- and breast cancer risk-associated SNPs, leading to 9 gene annotations: *ID4, ZMIZ1, MCHR1* (in GRAIL)/*MKL1* (in HapMap), *ESR1, RAD51B, TNS1, TNP1, TET2* and *FAM46A*. Of the 15 SNPs in height-colorectal cancer clusters, 8 SNPs were annotated to the same gene in pairs of height- and colorectal cancer-risk associated SNPs, leading to the following four gene annotations: *BMP2, PITX1, DCBLD1 and BARX1*. One prioritised cluster, cluster ID 22, contained two genes, *i*.*e. TNS1* and *TNP1*, to which height- and breast cancer risk-associated SNPs were annotated that were found associated in independent GWAS.

### Annotation of genomic region and regulatory function

According to Ensembl genome browser the majority of candidate SNPs (*n* = 48) are located in introns (*n* = 25) and in intergenic regions (*n* = 17) (Table 1). The remaining SNPs were located in an enhancer (*n* = 3), upstream of a gene (*n* = 3), the promotor (*n* = 3), an exon (*n* = 3), or the promotor flanking region (*n* = 1) (Table 1). According to RegulomeDB, 27 SNPs may affect transcription factor binding (score 1–5), of which five also affect the expression of a gene target, termed cis-eQTLs (score 1a–1f), and thus these had the highest regulatory evidence (Table 1).

### Pathway over-representation analyses

Pathway over-representation analysis based on the 26 gene annotations from GRAIL indicated the Indian hedgehog (Ihh) signalling pathway as the most significant overrepresented pathway (*p*-value = 7.78 × 10^−7^) (based on the following genes: *BMP2, STK36, IHH, PTCH1*) (Table 2). Pathways that followed were ligand-receptor interactions (*IHH, PTCH1*) (*p*-value = 5.76 × 10^−5^) and signalling in basal cell carcinoma (*BMP2, STK36, PTCH1*) (*p*-value = 6.73 × 10^−5^) (Table 2). For comparison, when using the 29 HapMap gene annotations, the most significant overrepresented pathways were the Ihh signalling pathway, signalling in basal cell carcinoma, and the Transforming Growth Factor-beta (TGF-β) signalling pathway (data not shown).

A separate pathway over-representation analysis for genes annotated to SNPs that were associated with height or post-menopausal breast cancer risk also retrieved the Ihh pathway as the most overrepresented pathway (*STK36, IHH*) (*p*-value = 1.13 × 10^−4^), as well as some distinct pathways, such as the ERBB4 signalling pathway (*ESR1, TNRC6B*) (*p*-value = 9.10 × 10^−3^) and androgen receptor pathway (*ESR1, ZMIZ1*) (*p*-value = 9.02 × 10^−3^) (Table 2). A separate pathway over-representation analysis for genes from clusters that contained SNPs associated with height or colorectal cancer risk, indicated that the Ihh signalling pathway (*BMP2, PTCH1*) (*p*-value = 2.81 × 10^−4^) and signalling in basal cell carcinoma (*BMP2, PTCH1*) (*p*-value = 2.53 × 10^−4^) (Table 2) were overrepresented.

### Gene ontology over-representation analyses

A gene ontology term over-representation analysis, based on the 26 gene annotations from GRAIL, indicated the following top three most significantly overrepresented gene ontology terms for molecular and biological processes: regulation of biosynthetic process (*p*-value = 4.85 × 10^−6^), regulation of macromolecule metabolic process (*p*-value = 2.85 × 10^−5^) and epithelial cell proliferation (*p*-value = 3.29 × 10^−5^) (Table 3).

## Discussion

We present a systematic approach for epidemiologic studies to prioritise SNPs associated with multiple complex diseases or traits using all GWAS repository data publically available to elucidate aetiologic pathways. The clustering methodology in this approach relies on the assumption that SNPs from GWAS found associated with complex diseases or traits are not randomly distributed across the genome, but tend to cluster in regions of low recombination^5^. This allows for a systematic narrowing down of the genomic search field and we were able to identify clusters that were of relevance to the height-cancer association. Twelve clusters were identified that contained at least one height- and one cancer risk-associated SNP annotated to the same gene. Height- and post-menopausal breast cancer risk-associated SNPs (*n* = 33) clustered together in 8 clusters. In these, 26 SNPs were annotated to the same gene in sets of two or more height- and breast cancer risk-associated SNPs, leading to the following 9 gene annotations: *ID4, ZMIZ1, MCHR1* (in GRAIL)/*MKL1* (in HapMap), *ESR1, RAD51B, TNS1, TNP1, TET2* and *FAM46A*. Height- and colorectal cancer risk-associated SNPs (*n* = 15) clustered together in four clusters. In these, 8 SNPs were annotated to the same gene in pairs of height- and colorectal cancer risk-associated SNPs, leading to the following four gene annotations: *BMP2, PITX1, DCBLD1*, and *BARX1*.

The SNP selection strategy proposed here can typically be used to identify shared mechanisms between multiple traits or diseases, using gene-environment interactions for example. A number of two-step methods have been developed based on genome-wide data prioritising relevant SNPs within the own study population and subsequently testing these SNPs for interactions^3^,^6^. These existing strategies prioritise SNPs related to exposure in cases and controls^23^ or SNPs related to the outcome^24^. The cocktail-method is an approach which combines features of two-step methods, the case-only design, and empirical Bayes techniques^25^. Still, these strategies inherently lead to a higher probability of type I error, because SNPs are prioritised based on a genome-wide scan in the own study population without replication of the result. This can be avoided by selecting SNPs from publically available GWAS data, independent of the own study population, and using the clustering methodology to identify genomic regions of importance in relation to the phenotypes of interest. For most SNP clusters marking these regions, there is no particular expectation that the set of SNPs associated with the phenotypes of interest are themselves causal variants. Rather, the clusters mark regions in the human genome, which correlate with one or more causal variants. Therefore, the GWAS SNPs found in a single region likely tag similar mechanisms or causal variants and, in a way, may act as replication of the same result. These SNPs can then be taken forward to test for gene-environment interactions. The SNPs in the clusters may collectively point to pathways explaining the link between height and cancer risk. Previously, Mendelian randomization has been employed to make causal inferences regarding the link between height and colorectal cancer risk utilising genetic variants as a proxy for height. For example, Thrift *et al*.^26^ suggested a causal association between height-increasing alleles and a higher colorectal cancer risk in women, but further investigation was warranted in men^26^. An additional advantage of the clustering approach is that it is also particularly suitable for the investigation of several SNPs at once, all within one cluster, *e*.*g*. through the use of a genetic risk score, thereby accounting for multiple SNP effects and reducing the multiple testing problem.

Our SNP selection approach may also have some limitations. For example, the size of the genomic sliding window affected the cluster size and the number of clusters identified. Also, the method is reliant on published GWAS data which are not freely available at *p*-values ≥ 1 × 10^−5^ in the NHGRI GWAS Catalog and *p*-values > 1 × 10^−3^ in the Johnson and O’Donnell database. Furthermore, the number of SNPs from GWAS on height is relatively high compared to the number of SNPs from GWAS on breast and colorectal cancer risk; this might have to do with the fact that anthropometric data such as height is available in most studies. Nevertheless, the observation that a number of pathways of relevance to both height, post-menopausal breast cancer risk, and colorectal cancer risk were found overrepresented among the genes annotated to the SNPs in the clusters suggests that this approach can reveal biologically relevant information.

The notion that specific genes^27^,^28^ and genetic variants^26^,^29^,^30^ may be relevant for explaining the height-cancer association has been suggested previously. Our systematic SNP selection strategy showed the Ihh signalling pathway to be overrepresented as based on variants that lie in/near *BMP2, IHH, PTCH1*, and *STK36*, when basing gene annotations on GRAIL. Cross-talks have been suggested between the Ihh signalling pathway and the Transforming Growth Factor-beta (TGF-β) signalling pathway, which was found in overrepresentation analyses using HapMap gene annotations. Both pathways are of relevance to processes in growth plate regulation and the length of bones^31^,^32^ as well as tumour development^33^,^34^. Few hypothesis-based candidate-gene studies have been performed on SNPs in Ihh signalling pathway genes and breast or colorectal cancer risk. SNPs in TGF-β signalling pathway genes have been associated with increased breast cancer risk^35^. Moreover, it has been found that a high number of at-risk variants in genes in the TGF-β signalling pathway increased the risk of colon and rectal cancer^36^. That cross-talks between Ihh and TGF-β signalling pathways are important in linking height to cancer, is likely when considering other complex diseases such as coronary artery disease (CAD). Consistent with an inverse association between height and CAD, a recent study showed that genetically determined height, as based on 180 height-associated SNPs from the Genetic Investigation of Anthropometric Traits (GIANT) consortium (which were not found in GWAS on CAD), was inversely associated with CAD, possibly via BMP/TGF-β signalling^37^. Furthermore, interestingly, the basal cell carcinoma pathway is also significantly overrepresented in our results, which supports the previously reported height-basal cell cancer association^38^.

A number of SNPs were annotated to genes that fall in unanticipated pathways. Even though these pathways were not identified in our pathway overrepresentation analysis, these SNPs may provide new clues about the mechanisms that influence growth in relation to adult-attained height and breast and colorectal cancer risk. For example, of interest may be the melanin-concentrating hormone receptor (*MCHR1*) gene, to which both height- and breast cancer risk-associated SNPs were annotated. Several studies have supported a role for *MCHR1* in the regulation of food consumption behaviour, energy expenditure and body weight^39^,^40^. Previously, a cross-sectional study found that polymorphisms in the *MCHR1* gene were associated with differences in body composition and interacted with energy-related lifestyle factors^41^. Body fatness is, next to adult-attained height, a convincing risk factor for post-menopausal breast cancer^7^. Therefore, nutrient-sensing processes might be a common mechanism linking height and other anthropometric factors to breast cancer risk.

Unexpectedly, no clusters were identified that contained SNPs that were associated with all three phenotypes, *i*.*e*. height, post-menopausal breast cancer risk, and colorectal cancer risk. This might be explained by the fact that the *p*-value cut-off (*p*-value = 1 × 10^−5^) used for GWAS SNPs, although liberal, was not sufficiently liberal to find clusters that represented all three phenotypes. Likely, at even more liberal *p*-values, there is a higher probability of finding a shared component to complex traits, such as height and the risk of cancer, which may be involving thousands of common alleles with rather small effects^42^. Our results suggest that, in addition to a shared component, there may also be different mechanisms through which height influences post-menopausal breast and colorectal cancer risk. The mechanisms identified linking height to colorectal cancer risk overlapped with those found in overall pathway overrepresentation analyses in this study and these may operate primarily through Ihh signalling. The mechanisms linking height to post-menopausal breast cancer risk may go through Ihh signalling as well as ERBB4 signalling and androgen receptor signalling. Both ERBB4 signalling^43^,^44^ and androgen receptor signalling^45^,^46^ are involved in mammary gland development. Future studies can utilise the SNPs in height-post-menopausal breast and height-colorectal cancer clusters to conduct mediation analyses between SNPs and specific cancer endpoints with height as a mediating factor or to perform interaction analyses between SNPs and height with specific cancer endpoints.

Finally, it is only fair to mention that our method is likely to pick up some degree of pleiotropic effects in terms of SNP effects or gene effects, especially considering our prioritisation step in which we prioritised clusters with at least one height- and one cancer risk-associated SNP. In this report, however, we focused on the instrumental value of the clusters in terms of future gene-environment interaction analyses or mediation analyses aimed at elucidating disease aetiology, rather than on trying to pinpoint pleiotropic SNPs or genes. Nevertheless, it is good to realise that several other methods exist that are aimed at identifying potential pleiotropic effects^47^,^48^,^49^. These methods may, in part, confirm the results at hand, when applied to the same topic. However, due to differences in input and methodology, it is likely that also different signals will be picked up. It is beyond the scope of this paper to identify all existing methods and validate these against each other, but we encourage future efforts in relation to this issue. Such efforts preferably need to include the use of simulated data in order to be able to draw conclusions about the extent to which different signals are picked up by different methods and about the extent to which different methods can distinguish between true signals and noise.

## Conclusion

We report a novel SNP selection approach to systematically restrict the number of SNPs for genotyping in large-scale studies aimed at elucidating aetiologic pathways. Our approach is of particular interest for studies with exhaustive bio-samples, in which a genome-wide approach is not feasible, and will reduce the costs of genotyping and the chance of false-positive findings. The SNPs identified can be used to, for example, study gene-environment interactions or to conduct mediation analyses. The novelty of this method is the comprehensive integration of publically available GWAS repositories on the basis of which SNPs associated with multiple linked complex traits and diseases can be identified as these are hypothesised to cluster in regions of low recombination. Such SNPs may serve as time-independent biomarkers of pathway involvement to mechanistically underpin established associations. Of interest in this paper was the association between adult-attained height and the risk of post-menopausal breast and colorectal cancer, for which the Ihh signalling pathway was found to be potentially important. This pathway was also found in separate analyses for height-post-menopausal breast cancer and height-colorectal cancer clusters, but there may also be different biological mechanisms through which height is associated with post-menopausal breast as compared to colorectal cancer risk.

## Additional Information

**How to cite this article**: Elands, R. J. J. *et al*. A systematic SNP selection approach to identify mechanisms underlying disease aetiology: linking height to post-menopausal breast and colorectal cancer risk. *Sci. Rep.* **7**, 41034; doi: 10.1038/srep41034 (2017).

**Publisher's note:** Springer Nature remains neutral with regard to jurisdictional claims in published maps and institutional affiliations.

## Supplementary Material

Supplementary Tables

## Figures and Tables

**Table 1:** Overview of the prioritised SNP clusters in which at least one height and one post-menopausal breast or colorectal cancer risk-associated SNP were annotated to the same gene as based on either HapMap or GRAIL, complemented by the SNP-annotation to biological regulatory function information and gene-annotation to enriched pathway and gene ontology categories.

Cluster ID	Genomic region based on Ensembl Genome Browser release 81			Chromosome and cytogenicbond based on Ensembl Genome Browser release 81	LD tag^c^				Mapped genein HapMap 37^g^	Annotated gene in GRAIL^h^	ConsensusPathDB analyses			
		GWAS catalogue				RegulomeDB					Gene ontology^i^			Pathway^j^
											SNP ID^a^	Phenotype^b^	Score^d^		Cis-eQTL^e^	Transcripition factor binding^f^	#1	#2	#3
**Cluster ID 22**	rs13387042	BC	intergenic	2q	0	0			*TNP1*	*TNP1*	✓	✓		
	rs2553026	H	enhancer		0	0			*TNP1*	NA				
	rs1351164	H	intron		0	0			*TNS1*	NA				
	rs16857609	BC	intron		0	5			*TNS1*	NA				
	rs6435999	H	intron		0	0			*TNS1*	*DIRC3*				
	rs3791950	H	intergenic		0	2b		*RUNX3, PAX5, TAF1*	*TNS1*	*TNS1*				
	rs10187066	H	intron		0	1 f	*SLC11A1, CYP27A1*		*ZNF142*	*STK36*				Hedgehog signalling
	rs12470505* a	H	upstream gene		1	1 f	*SLC23A3*	*USF1*	*CCDC108*	*IHH*	✓	✓	✓	Hedgehog signalling
	rs1052483*	H	exon		1	1 f	*SLC23A3*		*NHEJ1*	*IHH*	✓	✓	✓	Hedgehog signalling
	rs6724465*	H	intron		1	1 f	*SLC23A3*		*SLC23A3*	*IHH*	✓	✓	✓	Hedgehog signalling
	rs16859517	H	intergenic		0	5			*SLC23A3*	*IHH*	✓	✓	✓	Hedgehog signalling
**Cluster ID 27**	rs9790517	BC	intron	4q	0	0			*TET2*	*TET2*	✓	✓		
	rs10010325	H	intron		0	0			*TET2*	*TET2*	✓	✓		
	rs6855629	H	intron		0	6			*TET2*	*EEF1AL7*				
**Cluster ID 29**	rs526896* a	H	intergenic	5q	1	5			*PITX1*	*PITX1*	✓	✓		
	rs31198*	H	intron		1	5			*PITX1*	*PITX1*	✓	✓		
	rs647161	CRC	intron		0	5			*PITX1*	*PITX1*	✓	✓		
**Cluster ID 32**	rs1047014	H	upstream gene	6p	0	5			*ID4*	*ID4*	✓	✓		
	rs16882214	BC	intergenic		0	0			*ID4*	NA				
**Cluster ID 33**	rs2322633	H	intron	6q	0	6			*BCKDHB*	*BCKDHB*				
	rs310405	H	intergenic		0	0			*FAM46A*	NA				
	rs17530068	BC	intergenic		0	0			*FAM46A*	*FAM46A*				
**Cluster ID 34**	rs961764	H	intergenic	6q	0	0			*VGLL2*	*RFXDC1*				
	rs2057314	CRC	intron		0	4		*POLR2A, SPI1, TCF7L2, TCF12, NFIC, FOS*	*DCBLD1*	*DCBLD1*				
	rs9285425	H	intron		0	5			*DCBLD1*	*DCBLD1*				
**Cluster ID 7**	rs3757318* a	BC	intron	6q	1	2c		*HNF4A, HNF4G*	*C6orf97*	*C6orf97*				
	rs3734805*	BC	3 prime UTR variant		1	0			*C6orf97*	*C6orf97*				
	rs2046210	BC	intergenic		0	1 f	*C6orf97*		*ESR1*	*C6orf97*				
	rs9383938	BC	intron		0	5		*RFX3*	*ESR1*	*C6orf97*				
	rs543650	H	intron		0	0			*ESR1*	*ESR1*	✓	✓	✓	
	rs9383951	BC	intergenic		0	4		*GATA2, SETDB1*	*ESR1*	*ESR1*	✓	✓	✓	
	rs2982712	H	intron		0	0			*ESR1*	*ESR1*	✓	✓	✓	
**Cluster ID 39**	rs10114408	CRC	intergenic	9q	0	6			*BARX1*	*BARX1*	✓	✓		
	rs1257763	H	intergenic		0	0			*PTPDC1*	*BARX1*	✓	✓		
	rs16910061	H	upstream gene		0	5		*JUND*	*FBP2*	*FBP1*	✓	✓		
	rs473902	H	intron		0	3a		*POL2RA*	*PTCH1*	*PTCH1*	✓	✓	✓	Hedgehog signalling
	rs10512248	H	promotor		0	6			*PTCH1*	*PTCH1*	✓	✓	✓	Hedgehog signalling
	rs2025151	H	enhancer		0	1 f	*HABP4*	*POL2RA, RUNX3*	*ZNF367*	*HABP4*	✓	✓		
	rs10816533	H	promotor		0	1 f	*LOC642921*		*ZNF510*	*ZNF782*	✓	✓		
**Cluster ID 5**	rs704010	BC	intron	10q	0	2b		*POLR2A, SPI1, CTCF*	*ZMIZ1*	*ZMIZ1*	✓	✓		
	rs7916441* a	H	enhancer		1	5		*GABPB1*	*ZMIZ1*	*ZMIZ1*	✓	✓		
	rs780151*	H	intron		1	5			*ZMIZ1*	*ZMIZ1*	✓	✓		
	rs12355688	BC	exon		0	4		*USF1, USF2*	*ZMIZ1*	*ZMIZ1*	✓	✓		
	rs2145998*	H	intergenic		1	5			*PPIF*	*ZMIZ1*	✓	✓		
	rs941873 * a	H	promoter flanking		1	4		*HSF1, MAZ*	*ZCCHC24*	*ZMIZ1*	✓	✓		
**Cluster ID 15**	rs2588809	BC	intron	14q	0	0			*RAD51B*	*RAD51B*				
	rs1570106	H	intron		0	0			*RAD51B*	*RAD51B*				
	rs999737	BC	intron		0	6			*RAD51B*	*RAD51B*				
**Cluster ID 23**	rs961253	CRC	intergenic	20p	0	5			*FERMT1*	*FERMT1*			✓	
	rs967417*	H	intergenic		1	6			*BMP2*	*BMP2*	✓	✓	✓	Hedgehog signalling
	rs2145270*	H	intergenic		1	0			*BMP2*	*BMP2*	✓	✓	✓	Hedgehog signalling
	rs2145272* a	H	intergenic		1	3a		*STAT3, BCL11A, NFKB1, CHD2, EP300, IKZF1*	*BMP2*	*BMP2*	✓	✓	✓	Hedgehog signalling
	rs4813802	CRC	promotor		0	3a		*STAT3, CHD2, SETDB1, USF2, HNF4A, JUND, JUN, FOS, TRIM28, BACH1, TFAP2A, TFAP2C*	*BMP2*	*BMP2*	✓	✓	✓	Hedgehog signalling
**Cluster ID 25**	rs139909	H	intron	22q	0	2b		*RUNX3, BATF, FOXM1, NFIC, ATF2, MTA3*	*TNRC6B*	*TNRC6B*	✓	✓		
	rs5757949	H	intron		0	5			*MKL1*	*MCHR1*				
	rs6001930	BC	intron		0	5		*STAT3, CEBPB, EP300*	*MKL1*	*MCHR1*				

Abbreviations: eQTL; expression quantitative trait loci; GWAS, genome-wide association study; LD, linkage disequilibrium NA, data not available in GWAS catalogue; SNP, single nucleotide polymorphism.

^a^SNPs with the highest level of regulatory evidence were prioritised, indicated by the footnote (^a^). In cases were the regulatory evidence was equal, SNPs in high LD were prioritised according to the most significant *p*-value.

^b^Phenotype specifies whether a SNP derived from the GWAS catalogues by Hindorff *et al*.^14^ and Johnson O’Donnel *et al*.^15^ is associated with height (H), breast cancer risk (BC) or colorectal cancer risk (CRC).

^c^An LD tag equal to one denotes two or more SNPs within the same cluster that are in high LD (r^2^ > 0.7).

^d^RegulomeDB score for the putative regulatory function of a SNP.

^e^Genes for which the SNP is a cis-eQTL according to RegulomeDB. (Cis-eQTLs are SNPs that are associated with mRNA expression of (a) nearby located gene(s)).

^f^Known transcription factor proteins that are binding to the genomic coordinates of the SNP according to RegulomeDB.

^g^SNPs were annotated to a gene using the physical mapping of a SNP to a gene according to HapMap.

^h^Gene annotations using GRAIL (http://software.broadinstitute.org/mpg/grail/) were based on gene relationships among the complete set of SNPs listed in this table (S1 Table). In GRAIL, SNPs are annotated to genes by integrating the geographical location of a SNP derived from HapMap release 22with the biological data of a SNP obtained through text-mining using Pubmed 2014. GRAIL was set to correct for biases introduced by variable gene size when annotating the SNPs to genes. Large genes are more likely to have significant SNPs, and thus have a higher probability to be included in the regions that are being tested (Book: Computational Methods for Genetics of Complex Traits).

^i^Indicated with check-marks is whether the GRAIL gene annotation for a particular SNP contributed to the finding that the top three gene ontology terms, *i*.*e*. (#1) regulation of biosynthetic process (GO:009889), (#2) regulation of macromolecule metabolic process (GO:0060255), and (#3) epithelial cell proliferation (GO:0050673), were overrepresented in the total set of gene annotations from GRAIL (overrepresentation analyses were performed using ConsensusPathDB).

^j^Indicates whether a gene mapped to a SNP is annotated to the overrepresented Indian hedgehog signalling pathway according to ConsensusPathDB.

**Table 2:** Overrepresented pathways in prioritised SNP selection^a^.

Pathways	Set size	Number of genes from set in annotated gene list	Genes	*p*-value	*q*-value^b^	Pathway source
Overrepresented pathways using the genes annotated to the prioritised set of SNPs associated with height, post-menopausal breast and colorectal cancer risk.						
Hedgehog signalling pathway	52	4	*BMP2, STK36, IHH, PTCH1*	7.78 × 10^−7^	2.08 × 10^−5^	KEGG
Hedgehog signalling pathway	16	3	*STK36, IHH, PTCH1*	1.49 × 10^−6^	2.08 × 10^−5^	Wikipathways
Hedgehog	25	3	*STK36, IHH, PTCH1*	6.06 × 10^−6^	5.56 × 10^−5^	NetPath
Ligand-receptor interactions	8	2	*IHH, PTCH1*	5.76 × 10^−5^	3.77 × 10^−4^	Reactome
Basal cell carcinoma	55	3	*BMP2, STK36, PTCH1*	6.73 × 10^−5^	1.63 × 10^−3^	KEGG
HH-Core	19	2	*IHH, PTCH1*	3.48 × 10^−4^	1.63 × 10^−3^	Signalink
Signalling events mediated by the Hedgehog family	23	2	*IHH, PTCH1*	5.14 × 10^−4^	2.06 × 10^−3^	PID
Hedgehog, on, state	42	2	*IHH, PTCH1*	1.41 × 10^−3^	4.93 × 10^−3^	Reactome
Hedgehog signalling events mediated by Gli proteins	50	2	*STK36, PTCH1*	2.24 × 10^−3^	6.97 × 10^−3^	PID
Endochondral ossification	64	2	*IHH, PTCH1*	3.83 × 10^−3^	1.07 × 10^−3^	Wikipathways
TGF-beta signalling pathway	80	2	*BMP2, ID4*	5.96 × 10^−3^	1.48 × 10^−3^	KEGG
Signalling by Hedgehog	87	2	*IHH, PTCH1*	6.41 × 10^−3^	1.48 × 10^−3^	Reactome
Class B/2 (Secretin family receptors)	88	2	*IHH, PTCH1*	6.87 × 10^−3^	1.48 × 10^−3^	Reactome
Overrepresented pathways using the genes annotated to the prioritised SNPs associated with height and post-menopausal breast cancer risk.						
Hedgehog signalling pathway	16	2	*STK36, IHH*	1.13 × 10^−4^	1.35 × 10^−3^	Wikipathways
Hedgehog	25	2	*STK36, IHH*	2.81 × 10^−4^	1.68 × 10^−3^	NetPath
Hedgehog signalling pathway	52	2	*STK36, IHH*	1.18 × 10^−3^	4.70 × 10^−3^	KEGG
Signalling by *ERBB4*	153	2	*ESR1, TNRC6B*	9.02 × 10^−3^	2.19 × 10^−2^	Reactome
Androgen receptor	149	2	*ESR1, ZMIZ1*	9.14 × 10^−3^	2.19 × 10^−2^	NetPath
Overrepresented pathways using the genes annotated to the prioritised SNPs associated with height and colorectal cancer risk.						
Hedgehog signalling pathway	52	2	*BMP2, PTCH1*	2.81 × 10^−4^	6.34 × 10^−4^	KEGG
Basal cell carcinoma	55	2	*BMP2, PTCH1*	2.53 × 10^−4^	6.34 × 10^−4^	KEGG

Abbreviations: SNP, single nucleotide polymorphism.

^a^Overrepresented pathways were retrieved using the SNP-gene annotations from GRAIL.

^b^The *p*-values are corrected for multiple testing using the false discovery rate method and are shown as *q*-values.

**Table 3:** Top ten most significantly overrepresented gene-ontology terms in prioritised SNP selection^a^.

GO terms^a^	Set size	Number of genes from set in annotated gene list	*p*-value	*q*-value^b^	Sub-analysis: height and breast cancer risk^c^	Sub-analysis: height and colorectal cancer risk^c^
GO:0009889 regulation of biosynthetic process	4061	15	4.85 × 10^−6^	6.21 × 10^−4^	✓	
GO:0060255 regulation of macromolecule metabolic process	5358	16	2.85 × 10^−5^	1.80 × 10^−3^	✓	
GO:0050673 epithelial cell proliferation	323	5	3.29 × 10^−5^	3.30 × 10^−2^	✓	✓
GO:0048754 branching morphogenesis of an epithelial tube	170	4	4.55 × 10^−5^	1.80 × 10^−3^	✓	✓
GO:0090304 nucleic acid metabolic process	4893	15	5.61 × 10^−5^	1.80 × 10^−3^	✓	
GO:0016070 RNA metabolic process	4339	14	7.48 × 10^−5^	1.81 × 10^−3^	✓	
GO:0061138 morphogenesis of a branching epithelium	202	4	8.47 × 10^−5^	1.81 × 10^−3^	✓	
GO:0048732 gland development	407	5	9.38 × 10^−5^	3.30 × 10^−3^	✓	✓
GO:0060322 head development	678	6	10.40 × 10^−4^	3.30 × 10^−3^	✓	
GO:0001763 morphogenesis of a branching structure	213	4	10.50 × 10^−4^	3.30 × 10^−3^	✓	

Abbreviations GO, gene ontology; SNP, single nucleotide polymorphism.

^a^Overrepresentation analysis for GO terms were performed using using the SNP-gene annotations from GRAIL.

^b^The *p*-values are corrected for multiple testing using the false discovery rate method and are available as *q*-values.

^c^The check-mark indicates which of the top 10 GO-terms from the main GO overrepresentation analysis were also present in separate analyses for breast and colorectal cancer risk.
