# Supplementary material for: A systematic SNP selection approach to identify mechanisms underlying disease aetiology: linking height to post-menopausal breast and colorectal cancer risk
Source: Sci Rep. 2017 Jan 24;7:41034. doi: 10.1038/srep41034 (PMC5259777; doi:10.1038/srep41034)
Supplement: Supplementary Tables [file srep41034-s1.doc]

**Supplementary Data**

**A systematic SNP selection approach to identify mechanisms underlying disease aetiology: linking height to post-menopausal breast and colorectal cancer risk**

Rachel JJ Elands ^1*^, Colinda CJM Simons ^1^, Mona Riemenschneider ^2,3^, Aaron Isaacs,^4,5^ Leo J Schouten ^1^, Bas A Verhage ^1^, Kristel Van Steen ^6^, Roger W. L. Godschalk ^7^, Piet A van den Brandt ^1^, Monika Stoll ^2,5^, Matty P Weijenberg ^1^

^1^ Department of Epidemiology, GROW–School for Oncology and Developmental Biology, Maastricht University, Maastricht, the Netherlands

^2^ Institute of Human Genetics, Genetic Epidemiology, University of Münster, Münster, Germany

^3^ Department of Bioinformatics, Straubing Center of Science, Straubing, Germany

^4^ Department of Epidemiology, Genetic Epidemiology Unit, Erasmus Medical Center, Rotterdam

^5^ Department of Biochemistry, Maastricht Centre for Systems Biology (MaCSBio), CARIM–School for Cardiovascular Diseases, Maastricht University, Maastricht, the Netherlands
^6^ Department of Electrical Engineering and Computer Science, Montefiore Institute, University of Liège, Liège, Belgium

^7^ Department of Pharmacology and Toxicology, NUTRIM–School of Nutrition and Translational Research in Metabolism, Maastricht University, Maastricht, the Netherlands

| **Table S1.** SNP clusters formed using SNPs from the GWAS catalogues by Hindorff *et al*., 2009 [1] and Johnson O’Donnell *et al*., 2009 [2]; SNPs were associated with height, post-menopausal breast or colorectal cancer risk at a p-value < 10^-5^ and clusters were formed using a 1 mega base pair window and included at least one height- and one cancer risk-associated SNP | | | | | | | | | | |
| --- | --- | --- | --- | --- | --- | --- | --- | --- | --- | --- |
| GWAS catalogue | | | HapMap Gene^a^ | GRAIL Gene^b^ | Cluster ID | LD tag^c^ | Genomic region based on the Ensemble Browser | RegulomeDB | | |
| SNP ID | *P* value | Phenotype |  |  |  |  |  | Score^d^ | eQTL^e^ | Protein binding^f^ |
| rs11548323 | 2x10^-06^ | Breast cancer | *WASF2* | *FCN3* | 1 | 0 | 3 prime UTR | 4 |  | *CTCF* |
| rs11809207 | 6x10^-08^ | Height | *CATSPER4* | *CATSPER4* | 1 | 0 | Intron | 0 |  | *-* |
| rs7532866 | 3x10^-08^ | Height | *LIN28AP1* | *LIN28* | 1 | 0 | Intron | 6 |  | *-* |
| rs1387389 | 4x10^-06^ | Breast cancer | *PBX1* | *PBX1* | 2 | 0 | Intron | 5 |  | *hCPE-R* |
| rs6670655 | 3x10^-06^ | Height | *PBX1* | *PBX1* | 2 | 0 | Non coding transcript exon variant | 4 |  | *HNF4A* |
| rs1046934 | 2x10^-31^ | Height | *TSEN15* | *C1orf19* | 3 | 1 | Missense variant | 6 |  | *-* |
| rs10911251 | 9x10^-08^ | Colorectal cancer | *LAMC1* | *LAMC1* | 3 | 0 | Intron | 6 |  | *-* |
| rs2274432 | 8x10^-09^ | Height | *TSEN15* | *C1orf19* | 3 | 1 | Missense variant | 4 |  | *E2F1* |
| rs3814333 | 2x10^-13^ | Height | *COLGALT2* | *C1orf19* | 3 | 0 | Upstream gene variant | 5 |  | *CEBPB, CDX2* |
| rs756199 | 7x10^-06^ | Height | *COLGALT2* | *C1orf19* | 3 | 0 | Intron | 5 |  | *-* |
| rs10508468 | 7x10^-05^ | Breast cancer | *FRMD4A* | *FRMD4A* | 4 | 0 | Intron | 6 |  | *-* |
| rs7909670 | 3x10^-09^ | Height | *CCDC3* | *CAMK1D* | 4 | 0 | Intergenic | 0 |  | *-* |
| rs12355688 | 6x10^-06^ | Breast cancer | *ZMIZ1* | *ZMIZ1* | 5 | 0 | Non coding transcript exon variant | 4 |  | *USF2* |
| rs2145998 | 4x10^-13^ | Height | *PPIF* | *ZMIZ1* | 5 | 0 | Intergenic | 5 |  | *-* |
| rs704010 | 4x10^-09^ | Breast cancer | *ZMIZ1* | *ZMIZ1* | 5 | 0 | Intron | 2b |  | *CTCF* |
| rs780151 | 2x10^-09^ | Height | *ZMIZ1* | *ZMIZ1* | 5 | 1 | Intron | 5 |  | *-* |
| rs7916441^a^ | 6x10^-10^ | Height | *ZMIZ1* | *ZMIZ1* | 5 | 1 | Intron | 5 |  | *-* |
| rs941873 | 4x10^-07^ | Height | *ZCCHC24* | *ZMIZ1* | 5 | 0 | Intron | 4 |  | *HSF1* |
| rs10510102 | 2x10^-06^ | Breast cancer | *ATE1* | *NSMCE4A* | 6 | 0 | Intron | 1f | *ATE1* | *-* |
| rs10510126 | 7x10^-07^ | Breast cancer | *BUB3* | *HMX2* | 6 | 0 | Intergenic | 5 |  | *CREBBP* |
| rs10736303 | 9x10^-06^ | Breast cancer | *FGFR2* | *FGFR2* | 6 | 1 | Intron | 5 |  | *-* |
| rs1078806 | 2x10^-06^ | Breast cancer | *FGFR2* | *FGFR2* | 6 | 1 | Intron | 5 |  | *-* |
| rs11199914 | 2x10^-08^ | Breast cancer | *FGFR2* | *FGFR2* | 6 | 0 | Intergenic | 0 |  | *-* |
| rs11200014 | 1x10^-05^ | Breast cancer | *FGFR2* | *FGFR2* | 6 | 1 | Intron | 5 |  | *-* |
| rs1219642 | 3x10^-16^ | Breast cancer | *FGFR2* | *FGFR2* | 6 | 0 | Intron | 6 |  | *-* |
| rs1219648 | 1x10^-10^ | Breast cancer | *FGFR2* | *FGFR2* | 6 | 1 | Intron | 0 |  | *-* |
| rs2420946 | 2x10^-10^ | Breast cancer | *FGFR2* | *FGFR2* | 6 | 1 | Intron | 0 |  | *-* |
| rs2912774 | 3x10^-15^ | Breast cancer | *FGFR2* | *FGFR2* | 6 | 1 | Intron | 5 |  | *-* |
| rs2936870 | 5x10^-15^ | Breast cancer | *FGFR2* | *FGFR2* | 6 | 1 | Intron | 5 |  | *-* |
| rs2981575 | 1x10^-08^ | Breast cancer | *FGFR2* | *FGFR2* | 6 | 1 | Intron | 6 |  | *-* |
| rs2981578 | 1x10^-15^ | Breast cancer | *FGFR2* | *FGFR2* | 6 | 1 | Intron | 2b |  | *Oct-1* |
| rs2981579 | 2x10^-10^ | Breast cancer | *FGFR2* | *FGFR2* | 6 | 1 | Intron | 5 |  | *-* |
| rs2981582 | 2x10^-76^ | Breast cancer | *FGFR2* | *FGFR2* | 6 | 1 | Intron | 5 |  | *-* |
| rs3135718 | 7x10^-05^ | Breast cancer | *FGFR2* | *FGFR2* | 6 | 0 | Intron | 5 |  | *-* |
| rs3750817 | 8x10^-08^ | Breast cancer | *FGFR2* | *FGFR2* | 6 | 1 | Intron | 4 |  | *E2F1* |
| rs4752571 | 1x10^-05^ | Breast cancer | *FGFR2* | NA | 6 | 0 | Intron | 0 |  | *-* |
| rs6585827 | 2x10^-06^ | Height | *PLEKHA1* | *HTRA1* | 6 | 0 | Intron | 5 |  | *RFX3* |
| rs2107425 | 7x10^-06^ | Breast cancer | *MRPL23* | *MRPL23* | 7 | 0 | Intron | 4 |  | *CTCF* |
| rs2237886 | 2x10^-13^ | Height | *KCNQ1* | *KCNQ1* | 7 | 0 | Intron | 4 |  | *USF1* |
| rs3817198 | 2x10^-11^ | Breast cancer | *LSP1* | *LSP1* | 7 | 0 | Intron | 5 |  | *-* |
| rs909116 | 7x10^-07^ | Breast cancer | *TNNT3* | *LSP1* | 7 | 0 | Intron | 5 |  | *-* |
| rs3782089 | 4x10^-13^ | Height | *SSSCA1* | *OVOL1* | 8 | 0 | Non coding transcript exon variant | 5 |  | *-* |
| rs3903072 | 9x10^-12^ | Breast cancer | *SNX32* | *EFEMP2* | 8 | 0 | Intergenic | 5 |  | *-* |
| rs3824999 | 4x10^-10^ | Colorectal cancer | *POLD3* | *CHRDL2* | 9 | 0 | Intron | 2b |  | *AP-3* |
| rs606452 | 2x10^-09^ | Height | *SERPINH1* | *SERPINH1* | 9 | 1 | Intron | 5 |  | *-* |
| rs634552 | 4x10^-13^ | Height | *SERPINH1* | *SERPINH1* | 9 | 1 | Intron | 5 |  | *-* |
| rs11820646 | 1x10^-09^ | Breast cancer | *BARX2* | *BARX2* | 10 | 0 | Regulatory region variant | 4 |  | *USF1* |
| rs654723 | 4x10^-11^ | Height | *FLI1* | *FLI1* | 10 | 0 | Intron | 5 |  | *-* |
| rs7107217 | 5x10^-07^ | Breast cancer | *BARX2* | *BARX2* | 10 | 0 | Intergenic | 5 |  | *-* |
| rs10771399 | 2x10^-12^ | Breast cancer | *PTHLH* | *PTHLH* | 11 | 0 | Intergenic | 0 |  | *-* |
| rs10843164 | 6x10^-12^ | Height | *CCDC91* | *CCDC91* | 11 | 0 | Intron | 0 |  | *-* |
| rs2638953 | 7x10^17^ | Height | *CCDC91* | *CCDC91* | 11 | 0 | Intron | 0 |  | *-* |
| rs7313833 | 6x10^-06^ | Breast cancer | *PTHLH* | *PTHLH* | 11 | 0 | Intergenic | 0 |  | *-* |
| rs10492321 | 7x10^-11^ | Height | *SOCS2* | *SOCS2* | 12 | 1 | Downstream gene variant | 6 |  | *-* |
| rs10859563 | 3x10^-12^ | Height | *CRADD* | *CRADD* | 12 | 0 | Intron | 0 |  | *-* |
| rs11107116 | 1x10^-34^ | Height | *SOCS2* | *SOCS2* | 12 | 1 | Downstream gene variant | 6 |  | *-* |
| rs3825199 | 2x10^-07^ | Height | *SOCS2* | *SOCS2* | 12 | 1 | 3 prime UTR variant | 0 |  | *-* |
| rs4761470 | 6x10^-07^ | Breast cancer | *PLXNC1* | *PLXNC1* | 12 | 0 | Intron | 5 |  | *-* |
| rs11571833 | 5x10^-08^ | Breast cancer | *BRCA2* | *BRCA2* | 13 | 0 | Stop gained | 6 |  | *-* |
| rs718444 | 2x10^-10^ | Height | *PDS5B* | *N4BP2L1* | 13 | 1 | Intergenic | 6 |  | *-* |
| rs7332115 | 6x10^-10^ | Height | *PDS5B* | *N4BP2L1* | 13 | 1 | Intergenic | 6 |  | *-* |
| rs17104630 | 8x10^-06^ | Height | *NKX2-8* | *NKX2-8* | 14 | 0 | Downstream gene variant | 0 |  | *-* |
| rs2236007 | 2x10^-13^ | Breast cancer | *PAX9* | *PAX9* | 14 | 0 | Intron | 5 |  | *-* |
| rs1570106 | 8x10^-09^ | Height | *RAD51B* | *RAD51B* | 15 | 0 | Intron | 0 |  | *-* |
| rs2588809 | 1x10^-10^ | Breast cancer | *RAD51B* | *RAD51B* | 15 | 0 | Intron | 0 |  | *-* |
| rs999737 | 2x10^-07^ | Breast cancer | *RAD51B* | *RAD51B* | 15 | 0 | Intron | 6 |  | *-* |
| rs7153027 | 1x10^-10^ | Height | *TRIP11* | *FBLN5* | 16 | 1 | Intergenic | 6 |  | *-* |
| rs7155279 | 1x10^-10^ | Height | *TRIP11* | *FBLN5* | 16 | 1 | Intron | 6 |  | *-* |
| rs8007661 | 6x10^-10^ | Height | *TRIP11* | *FBLN5* | 16 | 1 | Intron | 0 |  | *-* |
| rs941764 | 4x10^-10^ | Breast cancer | *CCDC88C* | *SMEK1* | 16 | 0 | Intron | 4 |  | *NR3C1, CREBBP* |
| rs3884558 | 4x10^-06^ | Breast cancer | *RORA* | *RORA* | 17 | 0 | Intron | 0 |  | *-* |
| rs7178424 | 6x10^-09^ | Height | *C2CD4A* | *FAM148A* | 17 | 0 | Upstream gene variant | 2b |  | *STAT3, MAFK* |
| rs2072153 | 4x10^-08^ | Height | *ZNF652* | *ZNF652* | 18 | 0 | Intron | 6 |  | *-* |
| rs2075555 | 8x10^-08^ | Breast cancer | *COL1A1* | *COL1A1* | 18 | 0 | Intron | 4 |  | *POLR2A, CEBPB, JUN* |
| rs4605213 | 3x10^-08^ | Height | *MBTD1* | *NME2* | 18 | 0 | Intron | 2b |  | *p300, CTCF, HDAC2, ZNF263* |
| rs12449568 | 2x10^-06^ | Height | *ANKFN1* | *ANKFN1* | 19 | 0 | Intron | 0 |  | *-* |
| rs1549519 | 6x10^-09^ | Height | *TMEM100* | *TMEM100* | 19 | 0 | Intergenic | 6 |  | *-* |
| rs227724 | 7x10^-15^ | Height | *C17orf67* | *NOG* | 19 | 0 | Intergenic | 0 |  | *-* |
| rs4794665 | 1x10^-07^ | Height | *C17orf67* | *C17orf67* | 19 | 0 | Intergenic | 3a |  | *AIRE, GATA1, Foxa2* |
| rs6504950 | 2x10^-13^ | Breast cancer | *STXBP4* | *COX11* | 19 | 0 | Intron | 0 |  | *-* |
| rs11082671 | 2x10^-08^ | Height | *CTIF* | NA | 20 | 0 | Intron | 0 |  | *-* |
| rs12953717 | 9x10^-12^ | Colorectal cancer | *SMAD7* | *SMAD7* | 20 | 1 | Intron | 5 |  | *-* |
| rs1787200 | 1x10^-10^ | Height | *DYM* | *RPL17* | 20 | 0 | Intron | 6 |  | *Foxl1, Fokl1, Foxa2, Freac-3* |
| rs4464148 | 3x10^-08^ | Colorectal cancer | *SMAD7* | *SMAD7* | 20 | 0 | Intron | 4 |  | *EBF1* |
| rs4939827 | 1x10^-07^ | Colorectal cancer | *SMAD7* | *SMAD7* | 20 | 1 | Intron | 5 |  | *Elf-1, ELF3, PU.1, Spic* |
| rs8099594 | 3x10^-07^ | Height | *DYM* | *RPL17* | 20 | 0 | Upstream gene variant | 3a |  | *CTCF, TP53* |
| rs9967417 | 5x10^-09^ | Height | *DYM* | *RPL17* | 20 | 0 | Intron | 0 |  | *-* |
| rs2279008 | 3x10^-08^ | Height | *MYO9B* | *MYO9B* | 21 | 0 | Intron | 5 |  | *-* |
| rs8100241 | 4x10^-08^ | Breast cancer | *ANKLE1* | *C19orf62* | 21 | 0 | Missense variant | 4 |  | *GATA1, TAF1* |
| rs8170 | 2x10^-09^ | Breast cancer | *BABAM1* | *C19orf62* | 21 | 0 | Synonymous variant | 4 |  | *CEBPB* |
| rs10187066 | 2x10^-07^ | Height | ZNF142 | STK36 | 22 | 0 | Intron | 1f | SLC11A1, CYP27A1 |  |
| rs1052483 | 1x10^-06^ | Height | *NHEJ1* | *IHH* | 22 | 1 | Non coding transcript exon variant | 1f | *SLC23A3* | *-* |
| rs12470505 | 9x10^-12^ | Height | *CCDC108/IHH* | *IHH* | 22 | 1 | Upstream gene variant | 1f | *SLC23A3* | *USF1* |
| rs13387042 | 1x10^-13^ | Breast cancer | *TNP1* | *TNP1* | 22 | 0 | Intergenic | 0 |  | *-* |
| rs1351164 | 2x10^-14^ | Height | *TNS1* | NA | 22 | 0 | Intron | 0 |  | *-* |
| rs16857609 | 1x10^-15^ | Breast cancer | *TNS1* | NA | 22 | 0 | Intron | 5 |  | *-* |
| rs16859517 | 5x10^-06^ | Height | *SLC23A3* | *IHH* | 22 | 0 | Intergenic | 0 |  | *-* |
| rs2553026 | 6x10^-08^ | Height | *TNP1* | NA | 22 | 0 | Intron | 0 |  | *-* |
| rs3791950 | 2x10^-06^ | Height | *TNS1* | *TNS1* | 22 | 0 | Intron | 2b |  | *PAX5* |
| rs6435999 | 7x10^-07^ | Height | *TNS1* | *DIRC3* | 22 | 0 | Intergenic | 0 |  | *-* |
| rs6724465 | 2x10^-08^ | Height | *SLC23A3 / NHEJ1* | *IHH* | 22 | 1 | Intron | 1f | *SLC23A3* | *-* |
| rs2145270 | 5x10^-18^ | Height | *BMP2* | *BMP2* | 23 | 1 | Intergenic | 0 |  | *-* |
| rs2145272 | 2x10^-24^ | Height | *BMP2* | *BMP2* | 23 | 1 | Intergenic | 5 |  | *NFKB1, BATF* |
| rs4813802 | 7x10^-06^ | Colorectal cancer | *BMP2* | BMP2 | 23 | 0 | promotor flanking region | 4 |  | *STAT3, TRIM28, TFAP2A, TFAP2C, USF2, SETDB1, HNF4a* |
| rs961253 | 2x10^-10^ | Colorectal cancer | *FERMT1* | *FERMT1* | 23 | 0 | Intergenic | 5 |  | *-* |
| rs967417 | 2x10^-08^ | Height | *BMP2* | *BMP2* | 23 | 1 | Intergenic | 6 |  | *-* |
| rs1074683 | 1x10^-14^ | Height | *PXMP4* | *CBFA2T2* | 24 | 1 | Intron | 0 |  | *-* |
| rs2284378 | 1x10^-08^ | Breast cancer | *RALY* | *RALY* | 24 | 0 | Intron | 0 |  | *-* |
| rs7274811 | 6x10^-22^ | Height | *ZNF341* | *CBFA2T2* | 24 | 1 | Intron | 0 |  | *-* |
| rs139909 | 2x10^-07^ | Height | *TNRC6B* | *TNRC6B* | 25 | 0 | Intron | 2b |  | *BATF* |
| rs5757949 | 4x10^-06^ | Height | *MKL1* | *MCHR1* | 25 | 0 | Intron | 5 |  | *-* |
| rs6001930 | 2x10^-06^ | Breast cancer | *MKL1* | *MCHR1* | 25 | 0 | Intron | 0 |  | *-* |
| rs4552313 | 2x10^-06^ | Height | *CMC1* | *ZCWPW2* | 26 | 0 | Intron | 6 |  | *-* |
| rs4973768 | 2x10^-08^ | Breast cancer | *SLC4A7* | *NEK10* | 26 | 0 | 3 prime UTR variant | 6 |  | *-* |
| rs10010325 | 4x10^-11^ | Height | *TET2* | *TET2* | 27 | 0 | Intron | 0 |  | *-* |
| rs6855629 | 2x10^-08^ | Height | *TET2* | *EEF1AL7* | 27 | 0 | Intron | 6 |  | *-* |
| rs9790517 | 4x10^-08^ | Breast cancer | *TET2* | *TET2* | 27 | 0 | Intron | 0 |  | *-* |
| rs13177718 | 3x10^-08^ | Height | *FER* | *FER* | 28 | 0 | Intron | 6 |  | *-* |
| rs367615 | 4x10^-08^ | Colorectal cancer | *MAN2A1* | *PJA2* | 28 | 0 | Intergenic | 5 |  | *-* |
| rs31198 | 8x10^-06^ | Height | *PITX1* | *PITX1* | 29 | 0 | Intron | 5 |  | *-* |
| rs526896 | 2x10^-13^ | Height | *PITX1* | *PITX1* | 29 | 0 | Intergenic | 5 |  | *-* |
| rs647161 | 1x10^-10^ | Colorectal cancer | *PITX1* | *PITX1* | 29 | 0 | Intron | 6 |  | *-* |
| rs6879260 | 2x10^-09^ | Height | *GFPT2* | *GFPT2* | 30 | 0 | Intron | 4 |  | *USF1* |
| rs7711990 | 8x10^-05^ | Breast cancer | *BTNL8* | NA | 30 | 0 | Non coding transcript exon variant | 6 |  | *-* |
| rs204247 | 8x10^-09^ | Breast cancer | *RANBP9* | *RANBP9* | 31 | 0 | Intergenic | 6 |  | *-* |
| rs853356 | 3x10^-06^ | Height | *CD83* | *RNF182* | 31 | 0 | Intergenic | 0 |  | *-* |
| rs1047014 | 2x10^-13^ | Height | *ID4* | *ID4* | 32 | 0 | Upstream gene | 5 |  | *-* |
| rs16882214 | 2x10^-06^ | Breast cancer | *ID4* | NA | 32 | 0 | Intergenic | 0 |  | *-* |
| rs17530068 | 3x10^-06^ | Breast cancer | *FAM46A* | *FAM46A* | 33 | 0 | Intergenic | 0 |  | *-* |
| rs2322633 | 3x10^-09^ | Height | *BCKDHB* | *BCKDHB* | 33 | 0 | Intron | 6 |  | *-* |
| rs310405 | 1x10^-10^ | Height | *FAM46A* | NA | 33 | 0 | Intergenic | 0 |  | *-* |
| rs2057314 | 3x10^-06^ | Colorectal cancer | *DCBLD1* | *DCBLD1* | 34 | 0 | Intron | 4 |  | *SPI1* |
| rs9285425 | 2x10^-08^ | Height | *DCBLD1* | *DCBLD1* | 34 | 0 | Intron | 0 |  | *-* |
| rs961764 | 1x10^-11^ | Height | *VGLL2* | *RFXDC1* | 34 | 0 | Intergenic | 0 |  | *-* |
| rs1361108 | 9x10^-06^ | Height | *CENPW* | *C6orf173* | 35 | 1 | Intergenic | 5 |  | *-* |
| rs1490384 | 1x10^-16^ | Height | *CENPW* | *C6orf173* | 35 | 1 | Intergenic | 0 |  | *-* |
| rs1490388 | 6x10^-07^ | Height | *CENPW* | *C6orf173* | 35 | 1 | Intergenic | 0 |  | *-* |
| rs2180341 | 3x10^-08^ | Breast cancer | *RNF146* | *ECHDC1* | 35 | 0 | Intron | 0 |  | *-* |
| rs4549631 | 5x10^-13^ | Height | *CENPW* | *C6orf173* | 35 | 1 | Downstream gene variant | 0 |  | *-* |
| rs2046210 | 2x10^-15^ | Breast cancer | *CCDC170* | *C6orf97* | 36 | 0 | Intergenic | 1f | *C6orf97* | *-* |
| rs2982712 | 4x10^-10^ | Height | *ESR1* | *ESR1* | 36 | 0 | Intron | 0 |  | *-* |
| rs3734805 | 1x10^-07^ | Breast cancer | *CCDC170* | *C6orf97* | 36 | 1 | Intron | 0 |  | *-* |
| rs3757318 | 2x10^-21^ | Breast cancer | *CCDC170* | *C6orf97* | 36 | 1 | Intron | 4 |  | *HNF4A, HNF4G* |
| rs543650 | 1x10^-17^ | Height | *ESR1* | *ESR1* | 36 | 0 | Intron | 0 |  | *-* |
| rs9383938 | 2x10^-10^ | Breast cancer | *ESR1* | *C6orf97* | 36 | 0 | Intron | 5 |  | *RFX3* |
| rs9383951 | 2x10^-06^ | Breast cancer | *ESR1* | *ESR1* | 36 | 0 | Intron | 4 |  | *GATA2* |
| rs9365723 | 4x10^-06^ | Colorectal cancer | *SYNJ2* | *SYNJ2* | 37 | 0 | Intron | 5 |  | *-* |
| rs9456307 | 2x10^-09^ | Height | *TULP4* | *GTF2H5* | 37 | 0 | 3 prime UTR variant | 0 |  | *-* |
| rs2128382 | 8x10^-06^ | Colorectal cancer | *GSDMC* | *FAM49B* | 38 | 0 | Intergenic | 0 |  | *-* |
| rs6470764 | 2x10^-28^ | Height | *GSDMC* | *MLZE* | 38 | 0 | Enhancer region | 5 |  | *-* |
| rs10114408 | 3x10^-06^ | Colorectal cancer | *BARX1* | *BARX1* | 39 | 0 | Intergenic | 6 |  | *-* |
| rs10512248 | 4x10^-11^ | Height | *PTCH1* | *PTCH1* | 39 | 0 | Intron | 6 |  | *-* |
| rs10816533 | 2x10^-06^ | Height | *ZNF510* | *ZNF782* | 39 | 0 | Intron | 1f | *LOC642921* |  |
| rs1257763 | 1x10^-09^ | Height | *PTPDC1* | *BARX1* | 39 | 0 | Intergenic | 0 |  | *-* |
| rs16910061 | 3x10^-06^ | Height | *FBP2* | *FBP1* | 39 | 0 | Upstream gene variant | 5 |  | *JUND* |
| rs2025151 | 2x10^-12^ | Height | *ZNF367* | *HABP4* | 39 | 0 | Intron | 1f | *HABP4* | *POLR2A* |
| rs473902 | 2x10^-17^ | Height | *PTCH1* | *PTCH1* | 39 | 0 | Intron | 5 |  | *-* |
| rs10759243 | 1x10^-08^ | Breast cancer | *KLF4* | *KLF4* | 40 | 0 | Upstream gene variant | 0 |  | *-* |
| rs4743034 | 2x10^-08^ | Height | *ZNF462* | *ZNF462* | 40 | 0 | Intron | 5 |  | *-* |
| rs7027110 | 2x10^-13^ | Height | *ZNF462* | *ZNF462* | 40 | 0 | Intergenic | 0 |  | *-* |
| rs865686 | 1x10^-34^ | Breast cancer | *KLF4* | NA | 40 | 0 | Intergenic | 0 |  | *-* |
| Abbreviations: eQTL; expression Quantitative Trait Locus GRAIL, Gene Relationships Among Implicated Loci; GWAS, genome-wide association study; ID, identification number; LD, linkage disequilibrium; NA, not available in GWAS catalogue; SNP, single nucleotide polymorphism. References: 1. Hindorff LA, Sethupathy P, Junkins HA, Ramos EM, Mehta JP, Collins FS, et al. Potential etiologic and functional implications of genome-wide association loci for human diseases and traits. Proc Natl Acad Sci U S A. 2009;106(23):9362-7. doi: 10.1073/pnas.0903103106. PubMed PMID: 19474294; PubMed Central PMCID: PMC2687147. 2. Johnson AD, O'Donnell CJ. An open access database of genome-wide association results. BMC medical genetics. 2009;10:6. doi: 10.1186/1471-2350-10-6. PubMed PMID: 19161620; PubMed Central PMCID: PMC2639349.  ^a^ Gene annotations were based on the physical mapping of a SNP according to HapMap.  ^b^ Gene annotations using GRAIL were based on gene relationships among the complete set of SNPs listed in this table.  ^c^ An LD tag equal to one denotes that two or more SNPs within the same cluster are in high LD (r^2^ > 0.7). For the prioritised clusters, SNPs with the highest level of regulatory evidence were prioritised. In cases where the regulatory evidence was equal, SNPs were prioritised according to the most significant p-value for the tested association.  ^d^ The score denotes the scale from RegulomeDB, with scores 1a-1f denoting SNPs that were likely linked to the expression of a gene target (cis-eQTLs), scores 2-3 denoting SNPs that likely only affected protein binding, scores 4-6 denoting SNPs for which there was minimal binding evidence, and score 0 denoting SNPs for which no evidence was available.  ^e^ Gene target of cis-eQTL (cis-eQTLs are SNPs that are associated with the regulation of mRNA expression of a nearby located gene) .  ^f^ Evidence for transcription factor binding at the genomic coordinate of a SNP according to RegulomeDB. The number in brackets on the left hand-side of a transcription factor indicates the number of transcription factor binding proteins that are binding to the genomic coordinate of a SNP. | | | | | | | | | | |

| **Table S2.** Characteristics of SNPs within clusters that included at least one height-associated GWAS SNP and one post-menopausal breast or colorectal cancer risk-associated GWAS SNP annotated to the same gene based on either HapMap or GRAIL | | | | | | | | | | |
| --- | --- | --- | --- | --- | --- | --- | --- | --- | --- | --- |
| Cluster ID | SNP ID^a^ | Gene annotation^b^ | First author GWAS | Phenotype^c^ | *P-*value | OR for breast or colorectal cancer,  or beta-coefficient for each  unit increase or decrease in height [95% CI] | Ancestry^d^ | Risk allele^e^ | MAF non-CEU^f^ | MAF CEU^g^ |
| **Cluster ID 22** | rs13387042 | *TNP1* | Michailidou*et al.*, 2013 | Breast cancer | 2x10^-57^ | 1.14 [1.11-1.16] | European | 0.51 (A) |  | 0.43 (G) |
|  |  |  | Fletcher *et al.*, 2011 | Breast cancer | 2x10^-10^ | 1.16 [1.11-1.22] | European | 0.52 (A) |  | 0.43 (G) |
|  |  |  | Li *et al.*, 2010 | Breast cancer | 9x10^-06^ | 1.18 [1.10-1.27] | European | 0.53 (A) |  | 0.43 (G) |
|  |  |  | Turnbull *et al.*, 2010 | Breast cancer | 2x10^-10^ | 1.21 [1.14-1.29] | European | 0.49 (A) |  | 0.43 (G) |
|  |  |  | Thomas *et al.*, 2009 | Breast cancer | 2x10^-08^ | 1.25 (Het) [1.15-1.37] | European | 0.51 (A) |  | 0.43 (G) |
|  |  |  | Stacey *et al.*, 2007 | Breast cancer | 1x10^-13^ | 1.2 [1.14-1.26] | European | 0.50 (A) |  | 0.43 (G) |
|  | rs2553026 | *TNP1* | N'Diaye *et al.*, 2011 | Height | 6x10^-08^ | 0.056 [0.036-0.076] unit increase | African | 0.19 (A) | 0.23 (A) | 0.30 (G) |
|  | rs1351164 | *TNS1* | Lango Allen *et al.*, 2010 | Height | 2x10^-14^ | 0.034 [NA] unit increase | European | 0.79 (T) |  | 0.19 (C) |
|  | rs16857609 | *TNS1* | Michailidou*et al.*, 2013 | Breast cancer | 1x10^-15^ | 1.08 [1.06-1.10] | European | 0.26 (T) |  | 0.29 (T) |
|  | rs6435999 | *TNS1* | N'Diaye *et al.*, 2011 | Height | 7x10^-07^ | 0.041 [0.025-0.057] unit increase | African | 0.64 (A) | 0.30 (G) | 0.01 (G) |
|  | rs3791950 | *TNS1* | N'Diaye *et al.*, 2011 | Height | 2x10^-06^ | 0.061 [0.036-0.086] unit decrease | African | 0.89 (A) | 0.17 (C) | 0.46 (C) |
|  | rs10187066 | *ZNF142* | Lango Allen *et al.*, 2010 | Height | 2x10^-07^ | NA | European | NA |  | 0.36 (A) |
|  | rs12470505*^a^ | *CCDC108/IHH* | Lango *et al.*, 2010 | Height | 9x10^-12^ | 0.041 [NR] unit increase | European | 0.90 (T) |  | 0.09 (G) |
|  | rs1052483 | *NHEJ1* | Gudbjartsson *et al.*, 2008 | Height | 1x10^-06^ | 6.9 [4.16-9.64] % SD taller | European | 0.91 (G) |  | 0.09 (T) |
|  | rs6724465* | *SLC23A3/NHEJ1* | Weedon *et al.*, 2008 | Height | 2x10^-08^ | 0.06 [0.02-0.10] SD shorter - among males | European | 0.10 (A) |  | 0.09 (A) |
|  | rs16859517* | *SLC23A3* | Okada *et al.*, 2010 | Height | 5x10^-06^ | NA | European | NA |  | 0.04 (T) |
| **Cluster ID 27** | rs9790517 | *TET2* | Michailidou*et al.*, 2013 | Breast cancer | 4x10^-08^ | 1.05 [1.03-1.08] | European | 0.23 (T) |  | 0.21 (T) |
|  | rs10010325 | *TET2* | Lango Allen *et al.*, 2010 | Height | 4x10^-11^ | 0.024 [NA] unit increase | European | 0.49 (A) |  | 0.48 (A) |
|  | rs6855629 | *TET2* | Berndt *et al.*, 2013 | Height | 2x10^-08^ | 1.14 [NA] | European | 0.63 (G) |  | 0.39 (A) |
| **Cluster ID 29** | rs526896*^a^ | *PITX1* | Lango Allen *et al.*, 2010 | Height | 2x10^-13^ | 1.15 [NA] | European | 0.73 (T) |  | 0.29 (G) |
|  |  |  | Berndt *et al.*, 2013 | Height | 9x10^-10^ | 0.03 [NA] unit increase | European | 0.72 (T) |  | 0.29 (G) |
|  | rs31198* | *PITX1* | Gudbjartsson *et al.*, 2008 | Height | 8x10^-06^ | 4.8 [2.64-6.96] % SD taller | European | 0.75 (T) |  | 0.26 (C) |
|  | rs647161 | *PITX1* | Jia *et al.*, 2012 | Colorectal cancer | 1x10^-10^ | 1.11 [1.08-1.15] | European | 0.67 (A) |  | 0.32 (C) |
|  |  |  | Jia *et al.*, 2012 | Colorectal cancer | 4x10^-10^ | 1.17 [1.11-1.22] | East-Asian | 0.31 (A) | 0.30 (A) | 0.32 (C) |
| **Cluster ID 32** | rs1047014 | *ID4* | Lango Allen *et al.*, 2010 | Height | 2x10^-13^ | 0.032 [NA] unit decrease | European | 0.75 (T) |  | 0.28 (C) |
|  | rs16882214 | *ID4* | Rinella *et al.*, 2013 | Breast cancer | 2x10^-06^ | 1.43 [NA] | European | 0.81 (NA) |  | 0.14 (G) |
| **Cluster ID 33** | rs2322633 | *BCKDHB* | Berndt *et al.*, 2013 | Height | 3x10^-09^ | 1.12 [NA] | European | 0.50 (T) |  | 0.48 (C) |
|  | rs310405 | *FAM46A* | Berndt *et al.*, 2013 | Height | 1x10^-10^ | 1.14 [NA] | European | 0.52 (A) |  | 0.47 (G) |
|  |  |  | Lango Allen *et al.*, 2010 | Height | 2x10^-13^ | 0.026 [NA] unit increase | European | 0.52 (A) |  | 0.47 (G) |
|  | rs17530068 | *FAM46A* | Garcia-Closas *et al.*, 2013 | Breast cancer | 3x10^-06^ | 1.09 [1.05-1.13] | European | 0.24 (C) |  | 0.20 (C) |
|  |  |  | Michailidou*et al.*, 2013 | Breast cancer | 8x10^-09^ | 1.05 [1.03-1.08] | European | 0.22 (G) |  | 0.20 (C) |
|  |  |  | Siddiq *et al.*, 2012 | Breast cancer | 3x10^-07^ | 1.16 [1.10-1.23] | Mixed | 0.24 (C) | 0.18 (C) | 0.20 (C) |
| **Cluster ID 34** | rs961764 | *VGLL2* | Lango Allen *et al.*, 2010 | Height | 1x10^-11^ | 0.024 [NA] unit decrease | European | 0.42 (C) |  | 0.41 (C) |
|  | rs2057314 | *DCBLD1* | Peters *et al.*, 2012 | Colorectal cancer | 3x10^-06^ | 1.08 [1.04-1.11] | European | 0.496 (G) |  | 0.49 (A) |
|  | rs9285425 | *DCBLD1* | Berndt *et al.*, 2013 | Height | 2x10^-08^ | 1.14 [NA] | European | 0.50 (G) |  | 0.49 (G) |
| **Cluster ID 7** | rs3757318*^a^ | *C6orf97* | Purrington *et al.*, 2013 | Breast cancer | 9x10^-06^ | 1.33 [1.17-1.51] | European | NA |  | 0.08 (A) |
|  |  |  | Michailidou*et al.*, 2013 | Breast cancer | 2x10^-21^ | 1.16 [1.12-1.21] | European | 0.07 (A) |  | 0.08 (A) |
|  |  |  | Turnbull *et al.*, 2010 | Breast cancer | 3x10^-06^ | 1.30 [1.17-1.46] | European | 0.07 (A) |  | 0.08 (A) |
|  | rs3734805* | *C6orf97* | Fletcher *et al., 2011* | Breast cancer | 1x10^-07^ | 1.19 [1.11-1.27] | European | 0.08 (C) |  | 0.06 (C) |
|  | rs2046210 | *ESR1* | Garcia-Closas *et al., 2013* | Breast cancer | 5x10^-16^ | 1.15 [1.11-1.19] | European | 0.42 (A) |  | 0.29 (A) |
|  |  |  | Couch *et al.*, 2013 | Breast cancer | 5x10^-09^ | 1.28 [1.18-1.39] | European | 0.08 (C) |  | 0.29 (A) |
|  |  |  | Zheng *et al.*, 2009 | Breast cancer | 2x10^-15^ | 1.29 [1.21-1.37] | Asian | 0.37 (A) | 0.36 (A) | 0.29 (A) |
|  | rs9383938 | *ESR1* | Siddiq *et al.*, 2012 | Breast cancer | 2x10^-10^ | 1.28 [NA] | Mixed | NA (T) | 0.15 (T) | 0.06 (T) |
|  | rs543650 | *ESR1* | Lango Allen *et al.*, 2010 | Height | 1x10^-17^ | 0.034 [NA] unit decrease | European | 0.40 (T) |  | 0.39 (T) |
|  | rs9383951 | *ESR1* | Long *et al.*, 2012 | Breast cancer | 2x10^-6^ | 1.14 [1.08-1.19] | Asian | 0.90 (G) | 0.07 (C) | 0.01 (C) |
|  | rs2982712 | *ESR1* | Berndt *et al.*, 2013 | Height | 4x10^-10^ | 1.17 [NA] | European | 0.47 (C) |  | 0.47 (C) |
| **Cluster ID 39** | rs10114408 | *BARX1* | Jiao *et al.*, 2012 | Colorectal cancer | 3x10^-06^ | 1.37 [1.20-1.56] | European | 0.76 (NA) |  | 0.26 (T) |
|  | rs1257763 | *BARX1* | Lango Allen *et al.*, 2010 | Height | 1x10^-09^ | 0.069 [NA] unit increase | European | 0.04 (A) |  | 0.03 (A) |
|  | rs16910061 | *FBP1* | Kim *et al.*, 2009 | Height | 3x10^-06^ | 0.53 NA cm decrease | Korean | 0.14 (T) | 0.12 (A) | 0.04 (A) |
|  | rs473902 | *PTCH1* | Lango Allen *et al.*, 2010 | Height | 2x10^-17^ | 0.069 [NA] unit increase | European | 0.92 (T) |  | 0.09 (G) |
|  | rs10512248 | *PTCH1* | Weedon *et al.*, 2008 | Height | 4x10^-11^ | 0.05 [0.02-0.07] SD taller - among males | European | 0.31 (G) |  | 0.33 (G) |
|  | rs2025151 | *HABP4* | Berndt *et al.*, 2013 | Height | 2x10^-12^ | 1.22 [NA] | European | 0.18 (G) |  | 0.17 (G) |
|  | rs10816533 | *ZNF782* | Lei *et al.*, 2008 | Height | 2x10^-06^ | NA | Chinese | 0.29 (C) | 0.29 (C) | 0.03 (C) |
| **Cluster ID 5** | rs704010 | *ZMIZ1* | Michailidou *et al.*, 2013 | Breast cancer | 7x10^-22^ | 1.08 [1.06-1.10] | European | 0.38 (T) |  | 0.44 (T) |
|  |  |  | Turnbull *et al.*, 2010 | Breast cancer | 4x10^-09^ | 1.07 [1.03-1.11] | European | 0.39 (A) |  | 0.44 (T) |
|  | rs7916441* | *ZMIZ1* | Lango Allen *et al.*, 2010 | Height | 6x10^-10^ | NA | European | NA |  | 0.48 (C) |
|  | rs780151*^a^ | *ZMIZ1* | Berndt *et al.*, 2013 | Height | 2x10^-09^ | 1.13 [NA] | European | 0.57 (G) |  | 0.46 (A) |
|  | rs12355688 | *ZMIZ1* | Song *et al.*, 2013 | Breast cancer | 6x10^-06^ | 1.24 [1.13-1.36] | African | 0.22 (T) | 0.21 (T) | 0.05 (T) |
|  | rs2145998* | *PPIF* | Lango Allen *et al.*, 2010 | Height | 4x10^-13^ | 0.026 [NA] unit decrease | European | 0.49 (A) |  | 0.48 (A) |
|  | rs941873*^a^ | *ZCCHC24* | N'Diaye *et al.*, 2011 | Height | 4x10^-07^ | NA | African | 0.41 (A) | 0.42 (A) | 0.49 (A) |
| **Cluster ID 15** | rs2588809 | *RAD51B* | Michailidou*et al.*, 2013 | Breast cancer | 1x10^-10^ | 1.08 [1.05-1.11] | European | 0.16 (T) |  | 0.18 (T) |
|  | rs1570106 | *RAD51B* | Lango Allen *et al.*, 2010 | Height | 8x10^-09^ | 0.026 [NA] unit decrease | European | 0.20 (T) |  | 0.21 (T) |
|  | rs999737 | *RAD51B* | Michailidou*et al.*, 2013 | Breast cancer | 3x10^-19^ | 1.09 [1.06-1.11] | European | 0.77 (C) |  | 0.27 (T) |
|  |  |  | Thomas *et al.*, 2009 | Breast cancer | 2x10^-07^ | 1.06 (Het) [1.01-1.14] | European | 0.76 (C) |  | 0.27 (T) |
| **Cluster ID 23** | rs961253 | *FERMT1* | Houlston *et al.*, 2008 | Colorectal cancer | 2x10^-10^ | 1.12 [1.08-1.16] | European | 0.36 (A) |  | 0.41 (A) |
|  | rs967417* | *BMP2* | Gudbjartsson *et al.*, 2008 | Height | 2x10^-08^ | 4.3 [2.73-5.87] % SD taller | European | 0.53 (C) |  | 0.40 (A) |
|  | rs2145270 | *BMP2* | Berndt *et al.*, 2013 | Height | 5x10^-18^ | 1.2 [NA] | European | 0.37 (C) |  | 0.42 (C) |
|  | rs2145272*^a^ | *BMP2* | Lango Allen *et al.*, 2010 | Height | 2x10^-24^ | 0.039 [NA] unit decrease | European | 0.65 (A) |  | 0.41 (G) |
|  | rs4813802 | *BMP2* | Peters *et al.*, 2012 | Colorectal cancer | 7x10^-06^ | 1.1 [1.05-1.14] | European | 0.34 (G) |  | 0.35 (G) |
| **Cluster ID 25** | rs139909 | *TNRC6B* | Estrada *et al.*, 2009 | Height | 2x10^-07^ | 0.25 [0.03-0.47] cm increase | European | 0.68 (T) |  | 0.32 (C) |
|  | rs5757949 | *MKL1* | Estrada *et al.*, 2009 | Height | 4x10^-06^ | NA | European | NA (T) |  | 0.31 (C) |
|  | rs6001930 | *MKL1* | Garcia-Closas *et al.*, 2013 | Breast cancer | 2x10^-06^ | 1.14 [1.08-1.20] | European | 0.11 (C) |  | 0.09 (C) |
|  |  |  | Michailidou*et al.*, 2013 | Breast cancer | 9x10^-19^ | 1.12 [1.09-1.16] | European | 0.11 (C) |  | 0.09 (C) |
| *(Footnotes on the next page)* | | | | | | | | | | |

| Abbreviations: CEU, Utah residents with Northern and Western European ancestry; CI, confidence interval*;*GRAIL, Gene Relationships Among Implicated Loci; GWAS, genome-wide association study; Het, heterozygous genotype; ID, identification number; MAF*,* minor allele frequency*;* NA, not available in GWAS catalogue; OR, odds ratio*;* SD, standard deviation; SNP, single nucleotide polymorphism.  ^a^ SNPs within clusters that are in high LD with each other (r^2^ > 0.7) are indicated with a star. SNPs with the highest level of regulatory evidence were prioritised and are indicated by the footnote (^a^). In cases where the regulatory evidence was equal, SNPs in high LD were prioritised according to the most significant p-value.  ^b^ Gene annotations using GRAIL were based on gene relationships among the complete set of SNPs listed in this table.  ^c^ Phenotype specifies whether the GWAS SNP was associated with height, breast cancer risk or colorectal cancer risk.  ^d^ Ethnicity of the population in which the GWAS was conducted.  ^e^ Risk allele frequency in controls as reported in each individual GWAS study.  ^f^  Minor allele frequency in non-CEU populations for a given GWAS SNP according to data on population genetics in the 1000Genomes project as can be derived from the Ensembl Genome Browser.  ^g^ Minor allele frequency in CEU populations for a given GWAS SNP according to data on population genetics in the 1000Genomes project as can be derived from the Ensembl Genome Browser. |
| --- |

| **Table S3**. Overrepresented pathways-using the genes annotated to the SNPs in all identified SNP clusters (i.e. before the prioritisation step in which clusters were prioritised that included at least one height- and one post-menopausal breast or colorectal cancer risk-associated SNP annotated to the same gene) | | | | | |
| --- | --- | --- | --- | --- | --- |
| Pathway name^a^ | Set size | Number of genes from set in annotated gene list | *p*-value | *q*-value ^b^ | Pathway source |
| Homologous recombination | [28](http://cpdb.molgen.mpg.de/CPDB/showSetDetails?sp=p&st=0) | 3 | 3.8x10^-4^ | 4.6x10^-2^ | KEGG |
| Endoderm differentiation | [7](http://cpdb.molgen.mpg.de/CPDB/showSetDetails?sp=p&st=0)1 | 4 | 4.7x10^-4^ | 4.6x10^-2^ | Wikipathways |
| Homologous recombination | [28](http://cpdb.molgen.mpg.de/CPDB/showSetDetails?sp=p&st=0) | 2 | 1.9x10^-3^ | 9.1x10^-2^ | Wikipathways |
| Collagen biosynthesis and modifying enzymes | [68](http://cpdb.molgen.mpg.de/CPDB/showSetDetails?sp=p&st=3) | 3 | 4.1x10^-3^ | 9.1x10^-2^ | Reactome |
| Endochondral Ossification | [64](http://cpdb.molgen.mpg.de/CPDB/showSetDetails?sp=p&st=2) | 3 | 4.1x10^-3^ | 9.1x10^-2^ | Wikipathways |
| Signalling by BMP | [21](http://cpdb.molgen.mpg.de/CPDB/showSetDetails?sp=p&st=5) | 2 | 4.1x10^-3^ | 9.1x10^-2^ | Reactome |
| BMP Signalling Pathway | [21](http://cpdb.molgen.mpg.de/CPDB/showSetDetails?sp=p&st=4) | 2 | 5.1x10^-3^ | 9.1x10^-2^ | HumanCyc |
| Signalling pathways regulating pluripotency of stem cells | [142](http://cpdb.molgen.mpg.de/CPDB/showSetDetails?sp=p&st=6) | 4 | 5.8x10^-3^ | 9.1x10^-2^ | PID |
| Signalling events mediated by the Hedgehog family | [23](http://cpdb.molgen.mpg.de/CPDB/showSetDetails?sp=p&st=6) | 2 | 6.1x10^-3^ | 9.1x10^-2^ | PID |
| Regulation of nuclear SMAD2/3 signalling | [77](http://cpdb.molgen.mpg.de/CPDB/showSetDetails?sp=p&st=7) | 3 | 6.9x10^-3^ | 9.1x10^-2^ | PID |
| Abbreviations: BMP, bone morphogenetic protein; GO, gene ontology; KEGG, Kyoto Encyclopedia of Genes and Genomes; PID, Pathway Interaction Database; SMAD, a set of protein homologs of both the Drosophila protein, mothers against decapentaplegic (MAD) and the Caenorhabditis elegans protein “SMA” (from gene SMA for small body size); SNP, single nucleotide polymorphism.  ^a^ Overrrepresented pathways were retrieved using the SNP-gene annotations from GRAIL.  ^b^ The *p*-values are corrected for multiple testing using the false discovery rate method and are shown as *q*-values. | | | | | |

| **Table S4**. Top ten most significantly overrepresented ontology terms using the genes annotated to the SNPs in all identified SNP clusters (i.e. before the prioritisation step in which clusters were prioritised that included at least one height- and one post-menopausal breast or colorectal cancer risk-associated SNP annotated to the same gene) | | | | |
| --- | --- | --- | --- | --- |
| GO terms^a^ | Set size | Number of genes from set in annotated gene list | *p*-value ^b^ | *q*-value |
| GO:0009887   organ morphogenesis | [918](http://cpdb.molgen.mpg.de/CPDB/showSetDetails?sp=g&st=0) | [19](http://cpdb.molgen.mpg.de/CPDB/showSetDetails?sp=g&st=0) | 5.3x10^-8^ | 1.3x10^-5^ |
| GO:0060348   bone development | [176](http://cpdb.molgen.mpg.de/CPDB/showSetDetails?sp=g&st=1) | [8](http://cpdb.molgen.mpg.de/CPDB/showSetDetails?sp=g&st=1) | 2.0x10^-6^ | 2.4x10^-4^ |
| GO:0001501   skeletal system development | [488](http://cpdb.molgen.mpg.de/CPDB/showSetDetails?sp=g&st=2) | [12](http://cpdb.molgen.mpg.de/CPDB/showSetDetails?sp=g&st=2) | 3.4x10^-6^ | 1.9x10^-3^ |
| GO:0048732   gland development | [407](http://cpdb.molgen.mpg.de/CPDB/showSetDetails?sp=g&st=3) | [11](http://cpdb.molgen.mpg.de/CPDB/showSetDetails?sp=g&st=3) | 3.4x10^-6^ | 2.7x10^-4^ |
| GO:0009653   anatomical structure morphogenesis | [2484](http://cpdb.molgen.mpg.de/CPDB/showSetDetails?sp=g&st=4) | [28](http://cpdb.molgen.mpg.de/CPDB/showSetDetails?sp=g&st=4) | 8.0x10^-6^ | 4.8x10^-4^ |
| GO:0090304   nucleic acid metabolic process | [4893](http://cpdb.molgen.mpg.de/CPDB/showSetDetails?sp=g&st=5) | [42](http://cpdb.molgen.mpg.de/CPDB/showSetDetails?sp=g&st=5) | 1.9x10^-5^ | 2.1x10^-3^ |
| GO:0010467   gene expression | [5291](http://cpdb.molgen.mpg.de/CPDB/showSetDetails?sp=g&st=6) | [44](http://cpdb.molgen.mpg.de/CPDB/showSetDetails?sp=g&st=6) | 2.3x10^-5^ | 2.1x10^-3^ |
| GO:0060255   regulation of macromolecule metabolic process | [5358](http://cpdb.molgen.mpg.de/CPDB/showSetDetails?sp=g&st=7) | [44](http://cpdb.molgen.mpg.de/CPDB/showSetDetails?sp=g&st=7) | 3.9x10^-5^ | 2.1x10^-3^ |
| GO:0043433   negative regulation of sequence-specific DNA binding transcription factor activity | [128](http://cpdb.molgen.mpg.de/CPDB/showSetDetails?sp=g&st=8) | [6](http://cpdb.molgen.mpg.de/CPDB/showSetDetails?sp=g&st=8) | 3.3x10^-5^ | 2.1x10^-3^ |
| GO:0030154   cell differentiation | [3504](http://cpdb.molgen.mpg.de/CPDB/showSetDetails?sp=g&st=9) | [33](http://cpdb.molgen.mpg.de/CPDB/showSetDetails?sp=g&st=9) | 3.9x10^-5^ | 2.1x10^-3^ |
| Abbreviations: GO, gene ontology; SNP, single nucleotide polymorphism.  ^a^ Overrepresentation analysis for GO terms were performed using using the SNP-gene annotations from GRAIL.  ^b^ The *p*-values are corrected for multiple testing using the false discovery rate method and are available as *q*-values. | | | | |
